# Materia Medica and Therapeutics; Medical Chemistry; Toxicology and Legal Medicine

**Published:** 1860-07

**Authors:** Emil Fischer


### MATERIA MEDICA AND THERAPEUTICS; MEDICAL CHEMISTRY; TOXICOLOGY AND LEGAL MEDICINE.

BY EMIL FISCHER, M.D.

### MATERIA MEDICA AND THERAPEUTICS.

#### I.—*On Penghawar Djambi.* By DR. VINKE.

After reporting fourteen cases in which the hemorrhage from serious wounds or bleeding ulcers was promptly and permanently arrested by the application of penghawar, the author communicates the experiments made by him with a view to ascertain the *modus operandi* of this remedy. The treatise contains the following information:—

1. *On the phytography of penghawar* (*paleæ cibotii.*)—The specimen examined by the author had been to the greater part separated from the stipes of the fern, and consists of delicate filaments, half an inch to two inches long, which are very soft, flexible, and so light that they keep themselves floating in the air for a long time. The shortest ones are thicker, dark gray or blackish, and are present in penghawar but in small quantity. The longer filaments are silky, shining, tortuous, very delicate, and of golden, light-brown color. It weighs so little that six grains constitute a considerable mass—sufficient to arrest bleeding from an artery one line in diameter. It swims on water, but falls to the bottom of the vessel after about half a minute, as it absorbs water; it gives an empyreumatic odor on being heated, burns faintly on being brought in contact with the flame of a candle, and detonates under complete combustion, diffusing an odor like agaric. On microscopic examination, the author found that the filaments of penghawar have nothing in common with hair. They form band-like, flat processes with articulations; their breadth surpasses their thickness three times and more. The joints are dark brown, resemble those of the shave-grass, but have delicate, often ramified processes. The part between the articulations is two to four times longer than wide, either of uniform width, or, in the dried state, conical, smaller at one end, of yellow color, translucent, covered with violet granules, which, together with the processes of the joints, fall off on applying a weak solution of caustic potassa, but become more distinct on being soaked in ether. The base of the filaments is either smaller, with branchy processes, or thicker, surrounded by hairs; their upper end is drawn out into a transparent, needle-shaped tubule. Each filament forms a hollow sheath which is partitioned by transparent diaphragms at the articulations. The cavity of the filament easily fills itself with any kind of fluid; fine powders do not penetrate into uninjured joints. In a solution of sulphate of iron the filaments become blackish, nearly opaque, and very brittle; if they have been previously soaked in ether, they assume a dark-brown color in the above solution. By iodine and dilute muriatic acid the physical properties of penghawar are not changed. A solution of caustic potassa becomes dark, the filaments themselves assume a bright yellow color in it, are rendered very smooth and soft, in consequence of losing their granular cover and their processes. The author does not attach much importance to the chemical reaction of penghawar, and only states that it forms not a green (v. Bemmelen) but a dark violet, blackish precipitate with the salts of iron.

2. *Results of experiments on freshly abstracted blood, and on living individuals.*—All the experiments show that the hemostatic effect of penghawar depends upon the capillary attraction of the water, which "exceeds the force by which the water in living blood is held in combination." The coagulation of the blood (also of that which is freshly drawn) is the immediate consequence of the blood being deprived of its watery portion—a fact which is confirmed by comparative experiments with capillary glass-tubes. Penghawar, however, acts with a five-times greater rapidity. A circumstance which promotes the firm adhesion of the coagulum to the surface of the wound and the permanent occlusion of the orifices of the vessels, consists in the elasticity and delicacy of the filaments; on moderate pressure the latter penetrate into the finest interstices and apertures on the surface of the wound, and thus cause coagulation of the blood not only on the surface of the wound, but also in the interstices of the tissues next to it. But it is particularly by the following qualities that penghawar excels other hemostatics:—

(1) It arrests, quicker than any other pharmaceutical means, (agaric, sponge, bovista, etc.) parenchymatous, venous, or arterial hemorrhage, provided the diameter of the artery does not exceed one line and a half. [The Indians stop bleeding, also, from greater arteries with penghawar.] (2) It produces a coagulum even in cases where the blood has changed so much that it has nearly lost the property of coagulating, or where the walls of the vessels are so diseased that they are incapable of a plastic process, as, for instance, in carcinomatous and scorbutic ulcers. (3) Penghawar does not change the vitality of the wound or ulcer, and therefore does not exert an injurious influence upon the healing process.

Penghawar acts better when crumbled than if applied entire. It is to be kept in a dry place. Five grains are sufficient to arrest considerable hemorrhage; more than one scruple was never required. It is pressed for two or three minutes directly on the bleeding surface, after which, if possible, a compressive bandage or strips of adhesive plaster are applied over it, taking care not to draw the wound too much together. If the bleeding does not proceed from the whole surface of the wound, it is not necessary to fill out the entire cavity of the wound or ulcer with penghawar. The hemorrhage ceased more rapidly, if the author pressed the penghawar (in the form of a pencil) so upon the bleeding surface that the filaments were directed perpendicularly against it. The internal administration of penghawar, as recommended by Gaupp and others, is quite useless.—(*Med. Zeitung Russlands*, 42–43, 1859, and *Schmidt's Jahrbücher*, April, 1860.)

### II.—*On the Employment of Santonine in Nervous Affections of the Eye.* By M. MARTINI.

M. Flourens presented to the Academy of Sciences of Paris, at the meeting of the twelfth of March, the second edition of M. Martini's work "on the coloration of sight and urine produced by the administration of santonine."

M. Flourens stated, that of the two effects of santonine mentioned, the former was certainly the most curious. Most persons who take santonine see objects green, some blue, and others straw colored. In the present edition, M. Martini has added new cases, and quoted those which have been published in England and France. In this edition also will be found some trials of santonine in nervous affections of the eye.

A woman, aged sixty-three, was affected with imperfect vision of the left eye. No visible alteration could be detected when M. Martini saw her in March, 1859. The pupil, however, was sluggish and larger than on the right side; in the aqueous humor a whitish cloud could be detected; and the patient could hardly distinguish light from darkness. M. Martini resolved upon trying santonine, and the patient was given for five days from four to six grains daily. She then saw, four or five times in the course of the day, objects of a greenish yellow, even with the weak eye. Eight grains were now daily given, and besides seeing things colored, as stated before, she could discern the faces of the persons around her. On the twentieth and twenty-second of March she still saw everything yellow, and more distinctly than before. The administration of santonine was now given up, and the improvement remained stationary.

In another case, santonine was given for two days to a patient who was amaurotic on both sides; and after that time the retina was found more sensible to light than before.

A third case is also mentioned, in which the patient had lost the right eye, and was amaurotic of the left. He was given ten grains daily. In a week he could read some words written in large letters on the wall.—(*London Lancet*, May 12, 1860.)

III.—*Subcutaneous Injections of Sulphate of Atropine in Tetanus; Recovery.* By M. PESCHEUX.

M. Pescheux has communicated the following case to the Surgical Society of Paris: A woman had met with a scalp wound and a compound fracture of both bones of the leg, in consequence of the falling in of a chimney. For three weeks she progressed very satisfactorily, when tetanus set in with great violence. After almost every one of the usual remedies had been employed, M. Pescheux subcutaneously injected, along the spinal processes of the cervical vertebræ, a solution of sulphate of atropine in the proportion of one grain of the salt to one hundred of water. About two-thirds of Pravaz's syringe were used, when the patient was seized with the well-known symptoms of belladonna poisoning, which lasted about twelve hours. The tetanic manifestations, when these symptoms abated, were much less severe; and after another injection, made twenty-four hours afterwards, which, however, did not affect the patient so much as the first, she completely recovered.—(*Ibid.*)

IV.—*Employment of Glycerin in the Preparation of Sinapisms.* By M. GRIMAULT.

A simplified and extemporaneous mode of preparing a sinapism is indicated by M. Grimault in the following formula: Glycerin, ʒiij; starch, ʒijss; essence of mustard, gtt. x. A thin layer applied on any material will induce more energetic and more prompt revulsion than that produced by the best mustard. The mixture should be stirred before employing it; and if the glycerin is of good quality the essence of mustard undergoes no alteration.—(*Bull. de Thérap.*, tome viii. p. 262, and *Med. Times and Gazette*, April 28, 1860.)

### V.—*Pepsine in the Severe and Obstinate Vomiting of Pregnant Women.*

M. L. Corvisart has of late advocated the use of pepsine to allay the very dangerous symptoms connected with the uncontrollable vomiting of pregnant women; and it would appear that excellent results have already been obtained. In *L'Union Médicale* of the seventeenth instant, we find two remarkable cases, reported by M. Baudot, in which the first doses of pepsine immediately relieved the patients, who had been brought to a very low ebb by constant vomiting.—(*London Lancet*, April 28, 1860.)

### VI.—*Iodized Oil of Juniper-berries.* By DR. HELLER.

Dr. Heller, of Vienna, found the use of this solution preferable to the ointment of iodide of potassium, which shows no action on the system, except when it has become yellow or decomposed, and contains free iodine, and the tincture of iodine, which sometimes, in prolonged use, produces very disagreeable effects. Iodine is readily soluble in oil of juniper-berries, but has to be mixed with it cautiously, and in small quantities at a time, in order to avoid explosion. The solution is brown and loses its color by standing, according to Dr. Heller; though a solution of twenty grains of iodine in one ounce of oil which had been prepared for an experiment, in order to determine the percentage of solubility, has not become colorless after standing several months. The preparation must be considered a chemical compound, for there is no free iodine detected in it by solution of starch. It has, furthermore, not the offensive smell of iodine; it does not stain nor destroy the epidermis like the tincture of iodine; and after its application iodine can be found in the urine as well as in the saliva of the patient. It recommends itself by all these qualities to the patronage of physicians. (*Wittstein's Vierteljahrschrift*, vol. viii. p. 462.) Oil of lavender can also be substituted for the oil of juniper.—(*Am. Druggists' Circular*, May, 1860.)

### VII.—*On Indian Hemp.* By DR. FRONMULLER, of Fuerth.

Indian hemp has become known but lately. First welcomed as a new narcotic remedy, it seems to have been abandoned again on account of uncertain and weak effect. This is at least the case in Germany and France, and the causes of it are easily found. The contradictions of the authors are discouraging. Rigler, for instance, observed an accelerated circulation after the use of haschisch; Sigismund, a diminished frequency of the pulse; Ley found an exciting effect on the sexual organs; Scanzoni denies it; some saw contraction of the pupil, others not, etc.

It is further a fact, that the effect of hemp in the East is quite different from the effect it produces in Europe; what can be effected in Calcutta or Algeria with one or two grains, requires in Europe thirty or forty times as much. Two grains are considered at Cairo a large dose; with us, eight grains are the smallest dose. There is probably some ethereal ingredient in the Oriental hemp which is destroyed by transport. Many practitioners hesitated to increase the doses sufficiently for the desired operation. Having once employed the remedy, I made up my mind to ascertain the truth, and continued my experiments until they embraced one thousand cases. But before giving the result, I will shortly refer to the plant and its therapeutical exhibition.

The external appearance of Indian and European hemp is the same, as are their botanical characters. The differences described by many authors are probably due to local causes only; the only real difference is the quantity of narcotic resin which is secreted by particular glands—like the lupulin in the hop-plant. This resin exudes, in India, from the flowers, leaves, and stalks, and is known there by the name of *churrus.* Its quantity increases with the southern direction.

The hemp cultivated in Edinburgh by A. Christison, exhibited no churrus; plants collected in Regent's Park, by Ley, contained about one-tenth as much resin as their Oriental sisters. French and German hemp appears to be richer in resin; the hemp growing in Italy still more so. Greece and Asia Minor furnish quite a powerful product; but by far the strongest is from Persia and India, although even there variations are found according to the soil. The quality of the resin seems to be subject to a similar variation, showing the same difference as rhubarb, roses, and poppies do in different climates. All of them require the rays of an Asiatic sun for the full development of their powers.

Asia is evidently the native country of hemp. This is proven by direct statements, by its being frequently mentioned in the history of Oriental nations, and by the very derivation of the name: Cannabis, Kannabis, from the Kanub—Ganja of the Arabs. (Ainslie, Mat. Ind., vol. ii. p. 190.) From China, India, and Persia, the cultivation of hemp spread to Egypt, over Africa to Europe and America, the fibres and the oil recommending the plant.

Already Homer speaks (Odyssey, N., 220–230,) of the exhilarating effects; for this is undoubtedly meant by the *Nepenthes* which caused all sufferings to be forgotten. Helena received it from Egypt, and there hemp was long esteemed on account of its intoxicating quality. (Prosp. Alpin., de medic. Ægypt.) Even to this day hemp-wine is used by the Orientals for the purpose of intoxication. Herodotus mentions cloths made from hemp, and refers also to fumigations used in Scythia. Galenus was well acquainted with the narcotic effects of haschisch; and in a Chinese work, now in the great library of Paris, it is stated that a Chinese physician, Hoa-Tho, living A.D. 220–230, administered to patients he wanted to operate on, a medicine prepared from hemp, and made them in that way insensible. Rigler says a preparation of hemp has been used for the same purpose in India since times immemorial; it is there called *Esrar* —*i.e.* secret. During the Crusades, a Persian, Hassan Ben Ali, famous for the number and fanaticism of his soldiers, used haschisch to intoxicate and excite them, so that all over Asia Mohammedans as well as Christians trembled before them. These fellows—exterminated in 1256 by the Mongolian chief, Hulakan Khan—received, from their using haschisch, the name of Haschischin, Haschaschin. By perversion, that word changed into assassin.

For the sake of the narcotism alone, hemp is extensively used at present in a great part of Asia, Africa, and South America, either smoked, (in Algeria, Morocco, by the Hottentots and Brazilians,) or in the form of electuary, confection, tincture, pastiles, etc. In spite of the Crusades, this narcotic quality of hemp was not known in France until after the campaign in Egypt. England received the first notice of it through her physicians practicing in the East Indies. In Germany, Dr. Molwitz pointed out hemp, in 1818, as a substitute for opium; Hahnemann employed it against affections of the nervous system. Since 1830 a large amount of information, regarding especially the power and qualities of the Indian hemp, has been published, mostly by English and French physicians.

The few chemical analyses made of the hemp plant, show in it a gum, a bitter extractive matter, albumen, chlorophyl, ethereal oil, (about twelve drops in twenty-eight ounces,) and a resin, called by some *cannabin*, which appears to be the effective ingredient. No alkaloids have been found so far.

The more important preparations which have been employed are: for internal use, the powdered herb, in pills or powder; Oriental haschisch—a mixture of the foregoing with ginger and cinnamon, in different proportions, therefore not very reliable, may be imitated by mixing the powder of the leaves with sugar and mucilage of gum tragacanth, so as to form cakes—the resinous extract, best in pills; a tincture made by dissolving the extract in ten to twenty parts of alcohol, and an emulsion of the resin. Churrus, the pure exudation of the plant, is used like the extract, but rarely met with in commerce. Externally the resin is applied endermatically, or in the form of embrocation, with oils, ointments, chloroform, etc.; also in injections.

Physiological experiments on healthy persons, instituted by Landerer, Beron, Rech, Wolff, Judee, Schroff, myself and others, show more or less a disturbance in the digestive tract; affection of the nervous system, with convulsive movements and sudden shocks; congestion to the brain; confused ideas; excited imagination, with frequently changing pictures; torpor, and sleep—the cerebral symptoms being more constant, while the others vary to a great extent, sometimes nothing being mentioned but a few confused ideas, followed by sleep. Fumigations with haschisch produced in two consumptive patients, first, some excitement, then a short sleep, without any important narcotic symptoms afterwards.

The effects of hemp are combated by the same means as those of opium: vegetable acids, coffee, emetics, cold applications, leeches to the temples. Landerer recommends sea-water, Cayenne pepper, salted victuals, caviar, strong coffee, and, as an emetic, the sulphate of copper. At Vienna, lemonade and coffee proved of good service. O'Shaughnessy gave acids in large doses.

The long-continued use of Indian hemp induces prostration, dropsy, and sometimes a liability to sudden fits of mania, with great inclination to destroy and ruin. This, however, I think more ascribable to the alcohol mixed and taken with the haschisch, than to the latter. The cataleptic fits of the Indian jugglers are also explained through the agency of hemp, though it occasions such fits extremely seldom.

During the short time since its use as a medicine has become known in Europe, hemp has been extensively employed, and mostly as a calming, antispasmodic remedy. Its power to excite the action of the uterus has not been confirmed. It has been exhibited in the following diseases, enumerated according to the efficacy of the remedy :—

*Tetanus* and *trismus*. Professor O'Shaughnessy, in Calcutta; Dr. Motz, in Gerlachsheim; some practitioners in America; at the University Hospital of Würzburg; Dr. Skae, in Edinburgh. Saussure gave the tincture against *trismus neonatorum*.

*Cardialgia.* The experience of Dr. Wolff, of Berlin, in this regard is confirmed by my own experiments.

*Delirium tremens.* I obtained a good effect from the tincture.

*Gonorrhœa.* The acute form is greatly improved by the addition of hemp extract to an emulsion.

As an *antidote to strychnine*, hemp was administered by O'Shaughnessy and Ley.

In *acute bronchitis* it is recommended by Hamberg and Martins.

Against *ischias* it is also extolled by Hamberg.

Good effects have been observed in *rheumatism* by Inglis, O'Shaughnessy, and myself.

*Spasmodic retention of urine* yielded to hemp in the hands of Dr. Brenner von Telsach. Bryan praised the diuretic virtues of the tincture, having employed it in anasarca; Schuodart speaks likewise of a diuretic quality. I have been unable to detect it.

Corrigan prescribed the tincture against *chorea;* O'Shaughnessy, the hemp in *hydrophobia*, and the tincture in *cholera*. Mauthner was successful with the remedy against *hooping-cough ;* J. Murray against *prosopalgia*, while it proved of no effect in the same affection to Professor Siebold.

Hubert employed hemp against the plague; Landerer against infarctus of the liver and spleen; Wolff against cancer of the womb. I found it to avail nothing in epilepsy.

Moreau and others, judging from the exhilarating and exciting effect of the Eastern product, thought it useful against melancholia and other mental disorders; the reports of the results obtained do not, however, agree.

For the purpose of inducing contractions of the uterus, Churchill, Simpson, Christison, Gregor, and Maguire availed themselves of the tincture of hemp. Heywood, in New York, pronounced haschisch a powerful emmenagogue. The experiments made in Germany, by Scanzoni and others, gave little satisfaction in this regard.

As an external remedy, I have used hemp many hundred times to relieve local pains of an inflammatory as well as neuralgic character.

Judging from my experiments, I have to assign to the Indian hemp a place among the so-called hypnotic medicines, next to opium; its effects are less intense, and the excretions are not so much suppressed by it. Digestion is not disturbed, the appetite rather increased, sickness of the stomach seldom induced, congestions never. Hemp may consequently be employed in inflammatory conditions. It disturbs the expectoration far less than opium. The nervous system is also not so much affected. The whole effect of hemp being less violent, and producing a more natural sleep, without interfering with the actions of the internal organs, it is certainly often preferable to opium, although it is not equal to that drug in strength and reliability. An alternating course of opium and Indian hemp seems particularly adapted to those cases where opium alone fails in producing the desired effect.

The best form are small pills made from the spirituous extract, with a little of the powdered leaves. The smallest dose to produce sleep may be set down at eight grains; a rapid increase is frequently required.*

All the pretended effects of hemp on the skin, kidneys, and sexual organs amount practically to nothing.—(*Prager Vierteljahrschrift*, xvii., 1860, and *Cleveland Medical Gazette*, May, 1860.)

VIII.—*Cutaneous Eruptions following the Use of Iodine.* By DR. FISCHER, of Vienna.

Dr. Fischer has published, in the *Med. Wochenschrift*, an able paper, wherein he shows that the continued use of iodine may give rise to eruptions, which assume different forms. He has, in the numerous cases which have presented these eruptions, noticed the four following forms: First, the erythematous; secondly, the papular; thirdly, the nodulo-pustular; and fourthly, the eczematous. The author does not venture to account for these peculiar effects of the alkaline salts of iodine, nor has he come to a fixed opinion as to the doses which may produce them. It is, however, important that the facts should be given proper publicity, as iodine eruptions might be attributed to other causes.—(*Lancet*, Am. ed., June, 1860.)

IX.—*On Gelsemin.* By DR. B. KEITH.

In the *Medical Press* for January second, Dr. B. Keith, of New York, has the following upon gelsemin, (prepared from the root of *Gelseminum sempervirens*,) which article he says he has used daily for the last eight years: "For controlling fevers of every type and grade; to arrest hemorrhage from the lungs, stomach, bowels, uterus, and urinary organs; in dysentery and bowel complaints; in spermatorrhœa, amaurosis, deafness, catarrhal affections, and hay-fever, I have used the gelsemin successfully. A single half-grain has arrested hemorrhage from the lungs, when all other remedies known to me had failed. While experimenting with it to ascertain its power for arresting hemorrhage, I gave to a lady, who had been confined two days previously, one and a half grains during twenty-four hours, which amount completely arrested the hemorrhage. I administered two grains, during the course of thirty-six hours, to a lady who had been suffering from uterine hemorrhage for two months, and that small quantity completely stopped the flow. So effectual is it in this form of hemorrhage, that I consider it quite a specific. In dysentery and bowel complaints, I consider it the most valuable article in the materia medica. From one-tenth to one-eighth of a grain, administered after each discharge, will shortly stop all hemorrhage

* The extract, in common use in this city, could not be given in so large a dose without fear of the most serious consequences.

and traces of disease." * * * "In dry-coughs, dependent upon irritation of the throat, it is the most prompt agent I have ever used. In nausea and vomiting I have used it, many cases yielding to a single dose of one-fourth of a grain." —(*New York Monthly Review*, March, 1860.)

### X.—*Formula for Ozanam's Solution of Bromine.*

Dr. Ozanam's bromine water, which he has used with so great advantage in pseudomembranous affections, (vide *Med.-Chir. Review*, January, 1860, p. 161,) is prepared according to the following formula: Distilled water, 100 grammes, (3½ ounces;) bromide of potassium and bromine, of each 10 centigrammes, (1⅔ grains.) Of this solution one to five drops are given daily in a glass of water in croup and pseudomembranous angina; larger doses cause pain in the stomach, and vomiting.—(*Bullet. Thérap.*, lvi., 1859.)

### XI.—*Sulphate of Quinia in Acute Articular Rheumatism.* By DR LANGE.

Dr. Lange has found large doses of sulphate of quinia extremely useful in acute articular rheumatism. He prescribes five grains of the sulphate with one grain of digitalis, to be taken four times daily. Sixteen doses were generally sufficient to effect a complete cure.—(*Deutsche Klinik*, 49, and *Foriep's Notizen*, ii. 4, 1860.)

### XII.—*Tannin as Antidote to Strychnine.* By PROFESSOR KURZAK, of Vienna.

From want of a reliable antidote, the treatment in cases of poisoning by strychnine hitherto consisted principally in endeavoring to evacuate the poison, to combat the frightful spasmodic symptoms by narcotics, and to re-establish respiration, when it finally ceased, by artificial means. Donné proposed iodine, chlorine, and bromine as antidotes to strychnine; Garrod, Rand, Morson, and Falck recommended prepared animal charcoal; but the efficacy of these substances has been neither tested sufficiently by experiment nor proved by experience. The same is true in regard to tannin, and the astringent vegetables containing it, their infusions, decoctions, etc. Although they recommended themselves by the fact that tannin forms chemical compounds, insoluble in water, with strychnine and other poisonous alkaloids, it seemed very probable that these products might be redissolved in the stomach and intestines, and thus be rendered capable of absorption; the virtue of tannin as antidote to strychnine was, therefore, considered very doubtful.

With a view to subject this matter to a thorough examination, and to ascertain the efficacy of tannin in preventing and allaying the symptoms of poisoning by strychnine, Professor Kurzak made a series of experiments on rabbits and dogs. At the end of his interesting and highly important memoir, he states that the results of his investigation permit him to draw the following conclusions:—

1. *Tannin*, if administered in time, *is an* excellent *chemical antidote to strychnine.*

2. The doubt, whether the precipitate formed by tannin in a solution of strychnine, although insoluble in water, would not be redissolved by the gastric and intestinal juice, and the strychnine thus reobtain its poisonous properties, is solved by these experiments on rabbits and dogs in a complete and highly gratifying manner.

3. The successful results in dogs and rabbits justify the expectation that *tannin would suspend the poisonous action of strychnine also in man*, even in cases where the evacuation of the tannate of strychnine, formed in the stomach, could not be accomplished.

4. These experiments show that *twenty to twenty-five times the quantity of tannin is required in order to suspend the poisonous action of strychnine.* In cases of poisoning it will be, however, advisable to administer a relatively larger proportion, as a part of the antidote will be absorbed by the usual contents of the stomach, particularly by gelatin.

5. As tannin has proved to be an antidote to nitrate of strychnia, which is much more soluble in water, there is so much greater reason to hope that it will be successful in poisoning by pure strychnia which dissolves in water with great difficulty.

6. *The same successful result* is to be expected from its administration in poisoning by the hard and tough *nux vomica*, which imparts the poison to aqueous fluids, but gradually and not very rapidly.

7. Tannin is a so much more valuable antidote in poisoning by strychnine, as *galls* in which it is contained can be readily procured, and thus be administered without much loss of time. They are easily reduced to a powder, which is given mixed with water. Another advantage is obtained by the vomiting which it is liable to produce. In the mean time, an *infusion* or *decoction* of powdered galls may be prepared.

On an average, Turkish galls contain fifty, the Illyrian galls twenty per cent. of tannin. At least one drachm of the former and two drachms and a half of the latter are therefore required to neutralize one grain of strychnine introduced into the stomach, but in general, especially if there is vomiting, a much larger quantity should be administered.

8. Another readily obtained substance containing tannin is *Chinese tea*, the efficacy of which, in poisoning by strychnine, is confirmed by our experiments. But these experiments (VII. and VIII.) have also shown that, in a decoction of tea-leaves, we cannot count upon the whole amount of tannin contained in them. In poisoning by a larger dose, it would therefore be necessary to administer so large an amount of green tea that the antidote itself might produce poisonous effects. One decigramme (1·3 grain) of nitrate of strychnine requires, as our experiments prove, ten drachms (600 grains, 40 teaspoonfuls) of green tea, which, according to Peligot's analysis, contain about fifteen grains of caffein. Tea is therefore applicable only in poisoning by smaller doses, but may otherwise be useful as adjuvant.

9. The efficacy of *roasted coffee* as chemical antidote to strychnine seemed to be much inferior. The amount of *caffeo-tannic acid* contained in coffee is, according to Payen, 3·5 to 5·0 per cent. But our experiments (IX., X., XI.) show that the decoction evidently contains a much smaller quantity of undecomposed tannic acid than this percentage would justify us in assuming. The decoction of 180 grains of roasted Cuba coffee (being adequate to 200 grains of the raw coffee, which should contain at least six grains of tannic acid) produced, according to the ninth experiment, merely a delay and diminution of the poisonous effect of 0·13 grain of nitrate of strychnine. In the tenth and eleventh experiments, 300 grains of raw coffee, which weighed, after roasting, 267 and 264 grains, and should have contained at least nine grains of tannic acid, had furnished a decoction which, as antidote to 0·13 grain of strychnine, was nearly inert, only delaying the appearance of the symptoms for a little while.

10. From *unroasted coffee*, so inconsiderable an amount of tannin is extracted, by boiling, that the employment of its decoction for our purpose is out of question.

11. *Oak bark* (of Quercus robur and Q. pedunculata) contains, according to Gerber, 8·5 per cent. of tannic acid, and imparts it readily to aqueous fluids. It deserves attention in poisoning by strychnine so much the more, as it can be procured without much delay, especially in the country. What has been said about the administration of galls equally applies to the use of the powder and decoction of this bark.

12. On account of their frequent occurrence and the large amount of tannin they contain, we have to mention in this connection: *acorns* (from Quercus robur and Q. pedunculata) with 9 per cent., the *bark of the horse-chestnut* with 8 per cent., *willow bark* with 5½ per cent., and *the green hull of walnuts*. The *radix tormentillæ* (with 17 per cent.,) *rad. caryophyllatæ* (with 31 per cent.,) and *rad. bistortæ*, are still richer in tannin, but can rarely be procured without much loss of time.

13. The solubility of the precipitate, produced by tannin in a solution of strychnine, by *acetic*, *citric*, and *tartaric acid*, (vide experiments with the same,) show the necessity of avoiding vegetable acids during the treatment of poisoning by strychnine with tannic acid.

14. The same applies to the internal use of *alcohol* and *alcoholic remedies*.

15. The reported experiments on rabbits have sufficiently proved that more active voluntary movements excite the spasms usually produced by strychnia, even when they otherwise would not have made their appearance. In treating cases of poisoning by strychnine, *it is therefore highly important to prohibit as much as possible all voluntary movements, and to avoid violent excitement of any other kind.*—(*Zeitschrift der K. K. Gesellschaft der Aerzte zu Wien*, March 12, 1860.)

---

### MEDICAL CHEMISTRY.

### I.—*Mode of Estimating the Quantity of Albumen in Albuminous Fluids.* By DR. C. BOEDEKER.

Dr. C. Boedeker recommends a solution of ferrocyanuret of potassium for the purpose of estimating the quantity of albumen contained in albuminous fluids. One cubic centimetre of a solution of 1·309 gramme of ferrocyanuret of potassium (2 KCy, FeCy + 3 HO) in 1000 C. C. of water corresponds exactly to 10 milligrammes of albumen. The albuminous solution which is to be tested must be in a diluted state and strongly acid, or must be rendered so by the addition of acetic acid. Dr. Boedeker considers this method useful in estimating the quantity of albumen contained in urine.—(*Henle and Pfeufer's Zeitschrift*, v., and *Vierteljahrscrift für Prakt. Heilkunde*, i. 1860.)

II.—*On the Composition of the Cerebral Substance.* By M. MULLER.

The cerebral substance of man contains a small quantity of creatine; this alkaloid is wanting in the brain of the ox; the author suspects that in the latter, leucine or one of its homologues is present.

But one of these substances, as well as the other, contains a large quantity of lactic acid and a small amount of fatty acids of the general formula $C^nH^nO^4$.

They contain neither creatinine nor urea, cystine, taurine, or succinic acid.

The brain of the ox contains, moreover, a small quantity of uric acid and a large proportion of inosit.

In the course of his researches, M. Müller has met with a substance not combined with phosphorus, which had much analogy with the cerebric acid of M. Frémy, but differed from it by its centesimal composition.—(*Ann. der Chemie und Pharm.*, ciii. p. 131, and *Journal de Pharmacie*, April 1, 1860.)

---

### TOXICOLOGY AND LEGAL MEDICINE.

I.—*On Poisoning by Arsenic.* By DR. BLONDLOT.

Dr. Blondlot has communicated, in a paper to the Paris Academy of Sciences, a fact which may be highly valuable in cases of poisoning by arsenic. After numerous experiments, he has come to the conclusion that the slightest quantity of greasy matter in contact with arsenious acid will reduce its solubility to about one-twentieth of what it was before. This explains at once why, in certain judicial investigations, arsenic has been sought for in vain in the liquid portion of the food contained in the stomach, when the food partly consisted of fatty substances, such as broth, milk, etc. It likewise explains how arsenious acid, taken in powder, may sometimes have sojourned a long time in the stomach before it produced any deleterious effect, since in such cases its action was hindered by the presence of fatty substances. Jugglers have been seen swallowing arsenic with impunity, because, according to Dr. Blondlot, they had previously taken the precaution to drink milk and eat fat bacon. Hence it follows, that in cases of poisoning by arsenic, fatty substances may be administered as real antidotes, capable of suspending the action of the poison for a considerable time, until more radical means of effecting a cure can be applied.—(*Med. Times and Gazette*, February 11, 1860.)

II.—*On the Detection of Metallic Poisons by means of Electrolysis.* By PROFESSOR BLOXAM.

At the last meeting of the Chemical Society, Professor Bloxam, of King's College, gave the result of a very successful investigation of this subject, and showed that, by proper refinement in the method of operating, the process of electrolysis may become a certain and delicate means of detecting one or all of the metallic poisons, at least with but few exceptions.

In an examination for arsenic by this method, the metal is obtained in the form of arseniuretted hydrogen; the process is therefore very similar to that of Marsh, over which, indeed, it does not present any advantage in point of delicacy. Marsh's process, however, although it is capable of doing all that can be done by electrolysis with even greater delicacy, is open to several well-known objections, which have stood in the way of its practical adoption by toxicologists. The process of electrolysis does not involve the use of zinc, which is so difficult to obtain pure. It forms a general method for the detection of several metallic poisons at once, and the material tested is not destroyed or inconveniently contaminated, but may be used for another operation. When the arsenic, on the other hand, is present in the state of arsenic acid, it cannot, according to Professor Bloxam's experiments, be detected with certainty by electrolysis. It is consequently necessary to reduce the arsenic acid by means of sulphurous acid. This is an objection which does not apply to Marsh's process. In his earlier experiments, Mr. Bloxam made use of a U-tube, containing dilute sulphuric acid; the substance to be tested was introduced into one of the limbs, and a cork with a bent tube fitted to its mouth; two platinum plates, leading from the poles of a battery containing five cells of Grove's, were introduced into the two limbs, and the liberated hydrogen passed through the bent tube, which was heated by a lamp, when the arsenic, if present, was deposited.

The form of the apparatus ultimately adopted as being the most convenient consists of a two or three ounce bottle, the bottom of which has been cut off and replaced by a piece of vegetable parchment bound on with platinum wire. To the mouth of the bottle is fitted a cork with a bent tube and a piece of platinum wire, which passes through the cork and turns up beneath in the form of a hook. A slip of platinum then hooks on the end of the wire, and passes nearly to the bottom of the bottle; it forms the negative pole of the arrangement. The bottle stands in an ordinary test-glass, and the positive pole, also of platinum, stands in the glass. Dilute sulphuric acid is put into the bottle and also the glass, so as to stand to the same height in both vessels. The substance to be tested is introduced into the bottle, the cork adjusted, and the wires connected with five cells of Grove's battery; the heat of a spirit-lamp is applied to the bent tube, and in the course of a quarter of an hour a distinct mirror is obtained if arsenic is present. Standard solutions, containing respectively a tenth, a hundredth, and a thousandth of a grain of arsenious acid, were prepared and examined by this process, and in every case a successful result was obtained. These solutions were then mixed with organic substances, such as the ordinary articles of food, meat, eggs, milk, etc., and the resulting matter examined. It was got into solution by means of chlorate of potash and hydrochloric acid, and the resulting fluid evaporated down, by means of a water bath, to a thick syrupy liquid. The arsenic was thus obtained in the state of arsenic acid, which does not give a certain result by the electrolytic process. Some sulphurous acid was therefore added, and the mixture introduced into the bottle, after expelling the excess of sulphurous acid by evaporation; a drachm of alcohol was then poured over the surfaces and the process put into operation.

The author prefers to add this drachm of alcohol in every case, inasmuch as it not only allays the frothing, but also affords an additional indication of the presence of arsenic; for, when these two substances are present—the alcohol and arsenic—the gas which escapes at the open end of the bent tube possesses a very peculiar odor, resembling alkarsin. If a little sulphurous acid be present, it also furnishes an additional character indicative of arsenic, namely, a slight yellow deposit, consisting of sulphide of arsenic, close to the borders of the metallic mirror. In all these experiments, of which a great number were made, the thousandth of a grain of arsenious acid was readily detected.

The other metals which may be detected by this process are mercury, antimony, copper, and bismuth; lead is precluded by the sulphuric acid which is present. These are all precipitated in the metallic form upon the slip of platinum; and even in the case of antimony a mere trace of antimoniuretted hydrogen is formed, the metal being all deposited upon the negative pole. The mode of proceeding in these cases is precisely similar to that adopted for arsenic. When the operation is concluded, the slip of platinum is detached, washed, and the deposit dissolved off and tested in the usual manner. Thus, where an organic mixture has to be examined for arsenic, mercury, antimony, copper, and bismuth, it is prepared in the manner just described for arsenic, and the resulting liquid introduced into the bottle, the drachm of alcohol poured over the surface of the contents, the cork adjusted, and the battery connected. The heat of a spirit-lamp is applied to the bent tube, and the operation continued for about a quarter of an hour or twenty minutes, when, if arsenic is present, a metallic deposit, accompanied by some crystals of arsenious acid, will be formed in the tube, and the escaping gas will have the alkarsin-like odor. The piece of platinum in the bottle is next removed, washed, and boiled in yellow sulphide of ammonium. Antimony would be dissolved and might be obtained as sulphide by evaporating this solution to dryness. The other metals would still remain on the plate. It is next boiled in nitric acid containing a trace of hydrochloric acid, the solution evaporated to a small bulk, and an excess of ammonia added. Oxide of bismuth would be precipitated, together with whatever traces of platinum had been dissolved. The precipitate may be dissolved in H Cl, and tested by pouring into water, etc. The ammoniacal filtrate would contain the copper, indicated by its blue color, and the mercury. By boiling with H Cl, and a slip of copper, the latter would be separated in the metallic form.

It will be seen, therefore, that this process of electrolysis admits of being very readily engrafted into the regular course of analysis, inasmuch as it affords an easy means of examining an organic mixture for all the ordinary metallic poisons without so far injuring or destroying the material as to preclude the employment of other tests.—(*Pharmaceutical Journal*, January, 1860, p. 376.)

### III.—*On Poisoning by Strychnia.* By DR. SCHNEIDER.

The author gives the following report of a case of suicidal poisoning by strychnia:—

A young man, eighteen years of age, informed his mother that he had poisoned himself, and then went to bed. A short time afterwards violent convulsions took place, especially in the upper extremities; an emetic, some hot milk, white of egg, and an enema were administered without success, and for a quarter of an hour he seemed to suffer greatly. Death supervened in half an hour or three-quarters after the ingestion of the poison. Three or four hours after death the body was still hot, and muscular contractions were distinctly visible in the face, neck, and upper extremities.

*Autopsy forty-eight hours after death.*—Contraction of the fingers; rigidity of the toes; spinal region and extremities of a violet color; belly, penis, and scrotum, greenish; vessels of the cerebral envelope gorged with black blood; serous effusion under the arachnoid; ingestion of the substance of the brain, etc. The chemical analysis of the contents of the stomach verified the presence of the poison.

The author follows up this case by some remarks on the cases of poisoning by strychnia on record at present, seeking by this comparison to establish the characters of such poisoning.

He classifies—first, the convulsions and the tetanic symptoms, which indicate lesion of the spinal marrow; next, paralysis of the lungs and the state of asphyxia which supervenes on the spasmodic contractions; then the symptoms which point out gastro-intestinal inflammation, such as vomiting, a burning sensation in the throat, pain in the epigastrium, etc. Among the cadaveric signs are remarkable—first, the relaxation of the muscles immediately after death, then their general or partial rigidity, slow putrefaction, violet color of the skin, hyperæmia of the brain and spinal cord, lungs, and heart, with repletion of the venous system.—(*Deutsche Klinik*, and *Dublin Medical Press*, April 11, 1860.)

